# Associations of Diabetes Mellitus Status and Geriatric Nutritional Risk Index with the Gut Microbiota in Nursing-Home Residents

**DOI:** 10.3390/nu18121966

**Published:** 2026-06-18

**Authors:** Teresa Gisinger, Luise Bellach, Christina Fastl, Cátia Pacífico, Marion Nehr, Athanasios Makristathis, Alexandra Kautzky-Willer, Thomas E. Dorner

**Affiliations:** 1 Division of Endocrinology and Metabolism, Department of Medicine III, Medical University of Vienna, 1090 Vienna, Austriaalexandra.kautzky-willer@meduniwien.ac.at (A.K.-W.); 2 Academy of Ageing Research, Haus der Barmherzigkeit, 1160 Vienna, Austria; 3 Biome Diagnostics GmbH, 1200 Vienna, Austria; 4 Division of Clinical Microbiology, Department of Laboratory Medicine, Medical University of Vienna, 1090 Vienna, Austria; 5 Centre for Public Health, Department of Social and Preventive Medicine, Medical University of Vienna, 1090 Vienna, Austria

**Keywords:** microbiota diversity, diabetes mellitus, nutritional index, nursing homes

## Abstract

Background/Objectives: Malnutrition and diabetes mellitus (DM) have been linked to gut microbial perturbations, yet data are scarce for the aging population, especially in a nursing-home setting. As this group is generally at risk for malnutrition, we aimed to investigate the link between DM and gut microbial patterns in interaction with nutritional risk status in nursing-home residents. Methods: Stool samples were collected from 173 nursing-home residents (77.5% female, mean age 86 years) and were analyzed via 16S rRNA sequencing. Furthermore, the Geriatric Nutritional Risk Index (GNRI) was assessed and data on comorbidity status, anthropometric measurements, and medication were acquired. Results: Fifty-one residents had DM (mean HbA1c 6%). There were no DM-related differences in alpha diversity (observed richness: *p* = 0.733; Shannon index: *p* = 0.747). PERMANOVA revealed slight differences in beta diversity according to GNRI (R^2^ = 0.009, *p* = 0.032), but no significant differences when adding DM status. Differential abundance analysis showed *Clostridium_Clostridiaceae*, *Haemophilus*, *Actinomycetaceae* and *Micrococcaceae* as significantly decreased with DM, independent of age, sex, and BMI. No interaction between DM and the GNRI in microbial diversity or composition was found. Conclusions: We report malnutrition-related differences in beta diversity and diabetes-related microbial taxa differences in nursing-home residents. DM status did not influence the relationship between the GNRI and gut microbiota in this population.

## 1. Introduction

Lower Geriatric Nutritional Risk Index (GNRI) scores in patients with diabetes mellitus (DM) are strongly associated with worse clinical outcomes [1,2]. In older DM individuals, reduced GNRI correlates with impaired β-cell function and higher blood glucose levels, while patients with DM and a low GNRI have up to three-fold increased all-cause mortality risk [1,2]. A low GNRI is also linked to increased DM complications including retinopathy, nephropathy, neuropathy, and sarcopenia in this population [3,4,5]. In this context, emerging evidence highlights the potential role of the gut microbiome, which has been shown to differ between older adults with and without DM in both alpha and beta diversity [6,7,8]. Generally, alpha diversity is reduced in older individuals with DM compared with non-DM peers, indicating lower microbial richness and a decreased abundance of beneficial bacteria, particularly butyrate-producing genera such as *Faecalibacterium* and *Anaerostipes* [7,8]. Beta diversity also revealed significant compositional shifts, with DM individuals showing a tendency toward pro-inflammatory taxa and a reduced presence of short-chain fatty acid-producing bacteria [7,8]. These differences between DM and non-DM individuals are becoming more pronounced in later life [9,10]. Certain taxa, including *Bifidobacteriaceae* and *Collinsella*, appear more frequently in older adults with DM, whereas *Clostridiaceae* and *Peptostreptococcaceae* are more commonly observed in metabolically healthy older individuals [9,10]. Furthermore, nursing-home residents have lower microbial diversity indices than peers living independently in the community, yet in both cohorts, frailty and nutritional status are key modifiers [11]. Malnutrition in general has been shown to be linked to a less favorable gut microbial composition, marked by reductions in alpha diversity or in abundance of *Lachnospirae*, potentially leaving individuals at risk for colonization with less favorable microbial communities [12]. This is especially compounded by age, leading to further reductions in alpha diversity or decreased abundances of short-chain fatty acid-producing taxa [12,13,14].

A link between gut microbiome diversity and the GNRI, a clinical tool to assess geriatric patients at risk for malnutrition, is biologically plausible, as reduced alpha diversity has been associated with poorer metabolic and inflammatory status, both of which can negatively affect nutritional health [15,16]. However, current literature does not provide direct evidence explicitly connecting GNRI with microbiome diversity in older adults with DM. Existing findings nevertheless suggest that reduced microbial diversity may be associated with an increased risk of malnutrition and metabolic dysregulation in aging populations.

Therefore, there is a need to systematically investigate and compare how both DM and GNRI influence gut microbiota diversity and composition in adults of older age. Understanding this relationship could help clarify whether nutritional risk contributes to microbiota alterations and may provide a basis for targeted nutritional and microbiota-focused interventions in geriatric DM populations. Therefore, the present study aimed to investigate the associations of diabetes status and nutritional risk, assessed by the GNRI, with gut microbiome diversity and composition in nursing-home residents. We further explored whether nutritional risk modifies potential diabetes-associated microbiome patterns.

## 2. Materials and Methods

### 2.1. Study Population

In this study, participants living in nursing homes were investigated (n = 173, females 77.5%). The data were obtained from two different care homes in Wiener Neustadt and Horn, Lower Austria, between September and November 2024. These homes provide long-term care, medical support, and daily assistance for geriatric or chronically ill people who need intensive nursing. Both homes focus on creating a supportive, home-like environment that improves residents’ quality of life. Patients who have received antibiotic or antifungal therapy up to one month prior to study enrolment were excluded.

### 2.2. Data Acquisition

Clinical data including medical history were taken from the electronic charts. Stool samples were stored using the Fecal Swab Collection and Preservation System by Norgen Biotec, which contains a stool preservative that allows for storage at room temperature for more than two years according to the manufacturer [17]. Residents who were able to independently use the toilet were provided with disposable paper stool collection aids to prevent contact with toilet water or cleaning agents and facilitate sample collection. For residents unable to use the toilet independently, samples were obtained directly from incontinence pads/diapers by the nursing staff. The samples were stored at room temperature prior to analysis. The study protocol and all required documents were reviewed and approved by the institutional ethics advisory board of Haus der Barmherzigkeit (approval no. 20240625_4.1, 25 June 2024). Residents with cognitive impairment or reduced decision-making capacity were excluded from the study.

### 2.3. Covariates

DM was defined as glycated hemoglobin (HbA1c) ≥ 6.5%, a documented diagnosis of DM in the medical history, or current treatment with metformin and/or insulin use. In addition, participants with an HbA1c ≥ 6.5% who were receiving GLP-1 receptor agonists or SGLT2 inhibitors were classified as having diabetes. This information was taken from the electronic charts of the nursing-home residents. There was no difference between type 1 or 2 DM. 

Nutritional status was assessed using the GNRI, a validated indicator calculated from serum albumin levels (taken from electronic charts) and the ratio of present to ideal body weight (with measured data for body weight and height in the frame of the study) [18]. GNRI categories (risk and no risk) were used to classify participants according to nutritional risk. The GNRI was calculated only for participants with available serum albumin and body weight measurements. Participants with missing data required for GNRI calculation were excluded from GNRI-related analyses. Consequently, analyses adjusted for the GNRI were performed using complete-case data and included only individuals with available GNRI values.

### 2.4. DNA Extraction, Sequencing and Bioinformatics

In total, 400 µL of the stool suspension in the Fecal Swab Collection and Preservation System was centrifuged for 3 min at 15,000 rpm, and 200 µL of the supernatant was used for the DNA extraction by the innuPREP AniPath DNA/RNA Kit–KFFLX (Innuscreen GmbH, Berlin, Germany) on a KingFisher Flex instrument (Thermo Fischer Scientific, Waltham, MA, USA). The V3–V4 region of the bacterial 16S rRNA gene was amplified and sequenced using the MiSeq Reagent Kit v3 600 (Illumina, San Diego, CA, USA) according to the 16S Metagenomic Sequencing Library Preparation protocol (Illumina) on the MiSeq platform (Illumina). Sequencing reads were processed using QIIME 2 (v2024.10; [19]). Paired-end reads were imported and their quality was assessed using FastQC v0.12.1 [20] and MultiQC v1.28 [21]. Primer trimming was performed with cutadapt v4.9 [22] using the forward primer sequence CCTACGGGNGGCWGCAG and the reverse primer sequence GGACTACNVGGGTATCTAAT. Denoising was carried out with DADA2, applying truncation lengths of 270 bp and 212 bp to forward and reverse reads, respectively. Taxonomic assignment was performed using the classify-sklearn naïve Bayes classifier trained on the SILVA v138 reference database. Features classified as mitochondrial or chloroplast sequences were removed from both the representative sequence set and the feature table.

### 2.5. Statistical Analysis

For the baseline table, categorical variables were presented as counts and percentages, metric variables were either presented as means plus standard deviations or, in case of skewed data where log2-data transformations were not possible, as medians (including interquartile ranges). Significance tests were performed using the chi-squared test, *t*-test or Wilcox test.

Microbial analyses were performed using the microViz pipeline [23]. After quality control, denoising, and taxonomic assignment, the initial feature table contained 2749 features across 180 samples, comprising 7,927,953 total reads. Sequencing depth ranged from 7219 to 333,729 reads per sample (median 35,366 reads). For downstream analyses, taxa were filtered using the microViz package, retaining taxa present in at least 10% of samples (minimum 18 of 180 samples) at a detection threshold of 0.1 (min_prevalence = 0.1, prev_detection_threshold = 0.1). Following filtering, 484 taxa remained, corresponding to 17.6% of the original features while retaining 80.6% of all sequencing reads (6,388,024 reads). The final feature table therefore consisted of 484 taxa across 180 samples. Sequencing depth in the filtered dataset ranged from 7101 to 297,743 reads per sample, with a median of 27,766 reads (IQR 21,858–36,160). No rarefaction was performed. Alpha diversity, beta diversity, and differential abundance analyses were conducted on the filtered dataset. Differential abundance testing was performed using ANCOM-BC2, which accounts for differences in sequencing depth and compositionality without requiring rarefaction. A more detailed description of the microbial analysis workflow follows below. All statistical analyses were conducted using R version 4.4.2 and RStudio version 2024.12.1.

### 2.6. Alpha Diversity

Alpha diversity was calculated at the species level using the observed richness and Shannon indices. Group differences in alpha diversity by DM status were first assessed using Wilcoxon rank-sum tests. Multivariable linear regression models were then fitted to evaluate associations between alpha diversity and DM status, including an interaction term with GNRI category and adjusting for sex, body mass index (BMI), and age. Adjusted marginal means were estimated using the emmeans package to visualize model-based effects stratified by GNRI status [24]. In addition, type III ANOVA was performed to test interaction effects between DM status and GNRI category. The results were visualized using boxplots and jittered scatter overlays, as well as plots of adjusted estimated marginal means with 95% confidence intervals. For the analysis, the R package microViz (for diversity calculations, via ps_calc_richness) and tidyverse/dplyr (for data handling) were used [23].

### 2.7. Beta Diversity

Data were agglomerated at the genus level and beta diversity was assessed using a Bray–Curtis dissimilarity distance matrix, followed by principal coordinate analysis (PCoA). The ordination results were visualized using two-dimensional PCoA plots with samples colored by DM status. Furthermore, permutational analysis of variance (PERMANOVA) models adjusting for GNRI, nursing home, BMI, age and sex were performed using the vegan package [25]. Homogeneity of multivariate dispersion was assessed using the betadisper and permutest functions from the vegan package with 999 permutations.

### 2.8. Differential Abundance Analyses

Differential abundance analyses were performed at the genus level using the analysis of composition with bias correction 2 (ANCOM-BC2) package [26]. Models included DM status as the main exposure and incorporated an interaction term with GNRI category, while adjusting for sex, BMI, age, and nursing home. No random effects were specified. Multiple testing was controlled using the false discovery rate (FDR) method, and structural zeros and sensitivity to pseudo-counts were accounted for using the default ANCOM-BC2 procedures. Taxa significantly associated with DM status or the DM × GNRI interaction were identified based on FDR-adjusted q-values < 0.05 and the ANCOM-BC2 sensitivity criteria. The results were exported and further processed for visualization.

For visualization, compositional genus-level abundances were calculated and merged with sample metadata. Relative abundances of significantly different taxa were plotted using violin and boxplots with jittered individual observations on a log10 scale.

**Figure 1:**
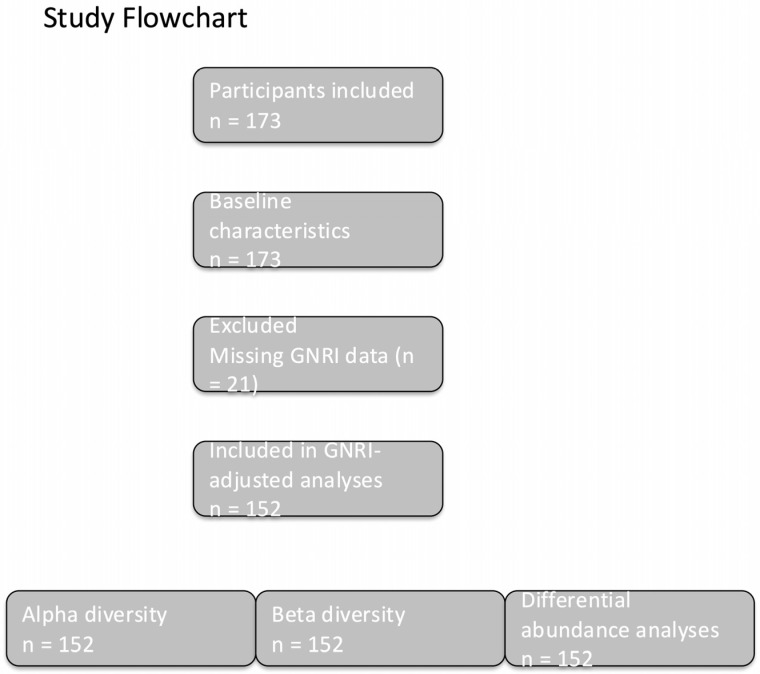
Study flowchart. A total of 173 nursing-home residents were included, comprising 122 participants without diabetes mellitus (DM) and 51 participants with DM. Baseline characteristics were available for all participants. GNRI data were missing for 21 individuals; therefore, GNRI-adjusted alpha diversity, beta diversity, and differential abundance analyses were performed in the remaining 152 participants.

The same differential abundance workflow was additionally applied at the family level. Analyses were also conducted with stratification by specific nursing home to account for potential clustering effects related to shared living environments. In Figure 1, we present the whole study flow.

## 3. Results

### 3.1. Baseline Characteristics

In Table 1, we report the baseline characteristics of individuals with and without DM. The mean age was very similar, with 83 years in people with DM and 85 years in people without DM (*p* = 0.200). In both cohorts, a higher rate of females was present, with 78% in non-DM and 75.89% in DM individuals (*p* = 0.900). Also, the HbA1c was slightly higher in people with DM (*p* = 0.002), and similar regarding most of the comorbidity frequencies. Regarding BMI, a higher rate of underweight can be seen in individuals without DM versus with DM.

### 3.2. Alpha Diversity

**Figure 2:**
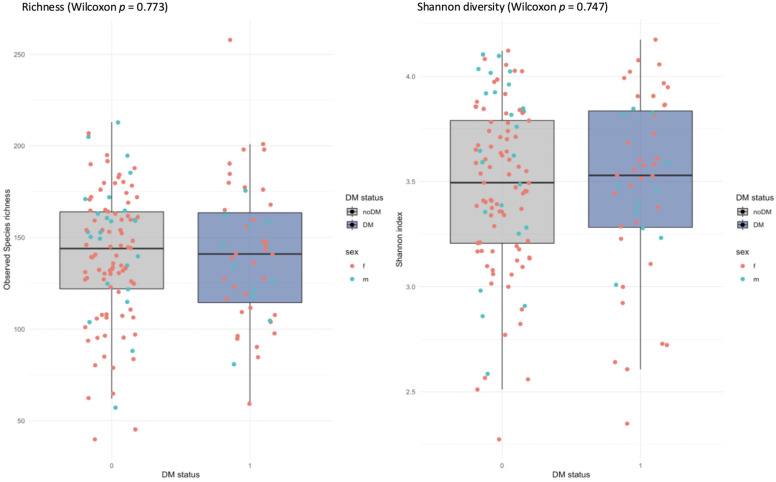
Comparison of alpha diversity between diabetes mellitus (DM) and NoDM groups. Boxplots show species richness (**left**) and Shannon diversity (**right**) stratified by DM status. Individual samples are overlaid as points and colored by sex (f = female, m = male). Differences between groups were assessed using the Wilcoxon rank-sum test, and corresponding *p*-values are indicated in the figure.

In the adjusted models, no differences in the observed richness (*p* = 0.773, Figure 2) or Shannon index (*p* = 0.747) were observed between the DM and noDM cohorts.

**Figure 3:**
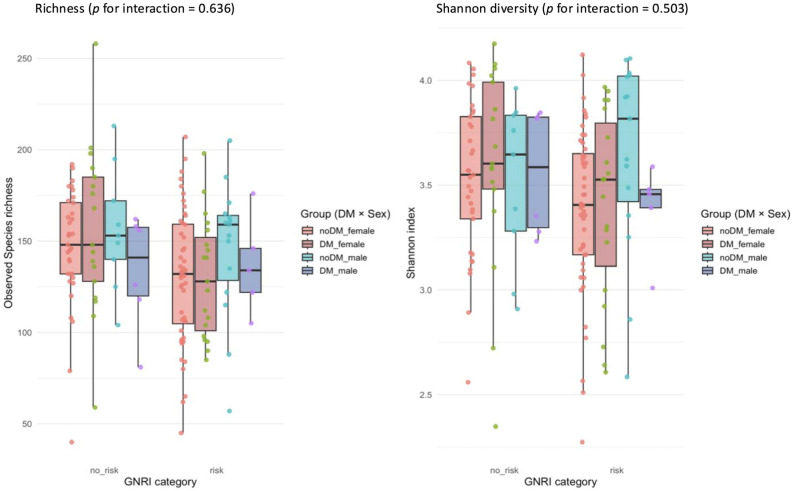
Association between diabetes mellitus (DM) status and alpha diversity across Geriatric Nutritional Risk Index (GNRI) categories. Boxplots show species richness (**left**) and Shannon diversity (**right**), stratified by GNRI category and DM status. Individual samples are overlaid as points and divided by sex. *p*-values represent the interaction between DM status and GNRI category derived from multivariable linear models adjusted for age, sex, and BMI.

Furthermore, a multiple linear regression model was used to examine associations between DM status, GNRI risk category, sex, BMI, age, and observed genus richness (Figure 3). The interaction between DM status and GNRI category on richness was not significant (B = −6.12, *p* = 0.636, CI95% −31.57–19.34). The same multiple linear regression model was used to assess the associations between DM status, GNRI risk category, sex, BMI, and age with Shannon diversity at the genus level. The interaction between DM status and GNRI category on Shannon diversity was also not significant (B = −0.10, *p* = 0.503, CI95% −0.39–0.19).

### 3.3. Beta Diversity

**Figure 4:**
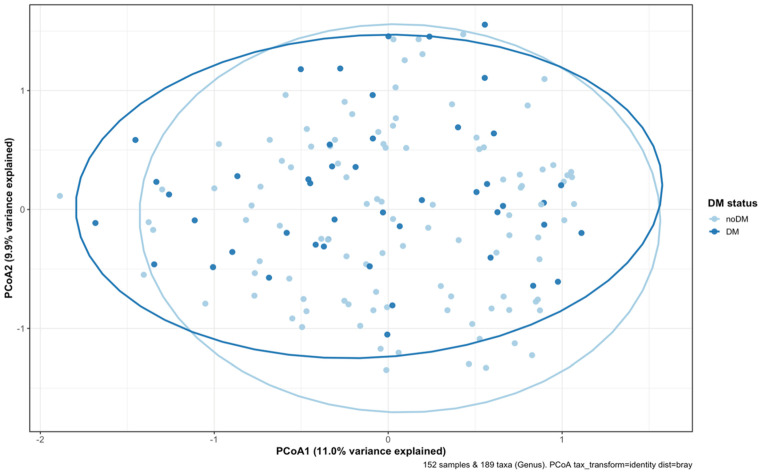
Principal coordinates analysis (PCoA) of Bray–Curtis dissimilarities at the genus level according to diabetes status. The first two axes explained 11.0% and 9.9% of the variance, respectively. Ellipses represent 95% confidence regions. Considerable overlap between groups was observed, indicating only limited separation of microbial community composition by diabetes status.

In PERMANOVA analyses based on Bray–Curtis distances, nutritional status (GNRI category) was significantly associated with differences in microbial community composition (R^2^ = 0.011, *p* = 0.032). DM status was not significantly associated with beta diversity after adjustment for GNRI, age, BMI, sex, and residence (R^2^ = 0.009, *p* = 0.116). This could also be seen by only a slight clustering tendency in the PCoA (Figure 4). No significant differences in multivariate dispersion were observed between participants with and without DM (permutest, *p* = 0.641).

### 3.4. Differential Abundance Analysis

**Figure 5:**
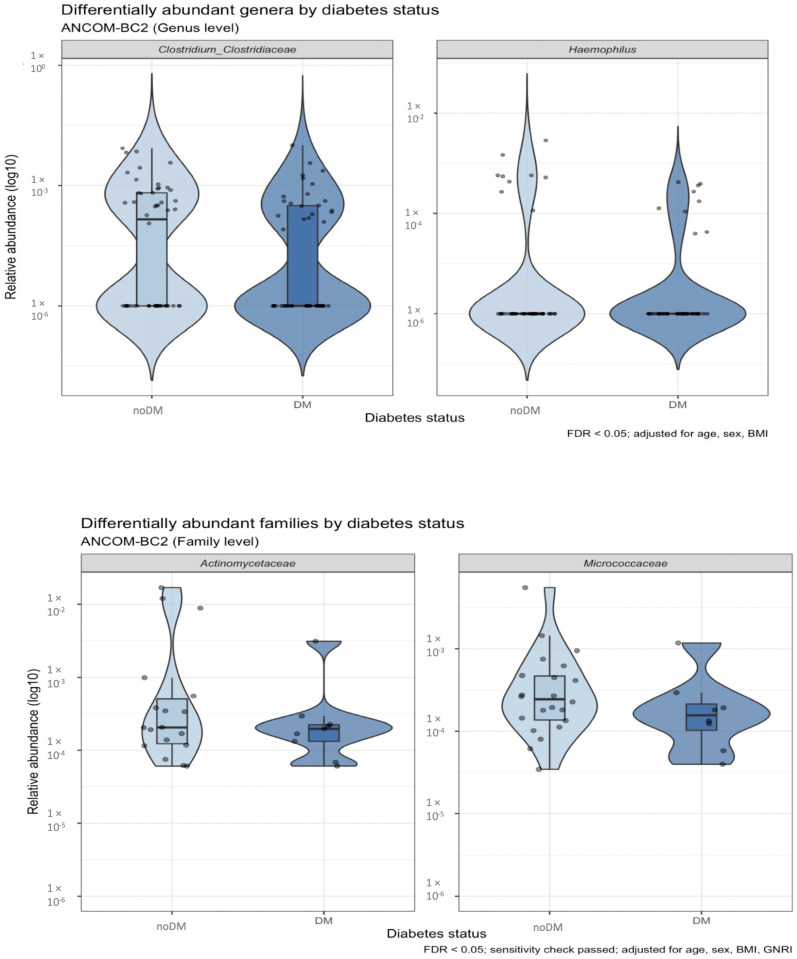
Differentially abundant bacterial taxa between individuals with and without diabetes mellitus (DM) identified by ANCOM-BC2 at the genus and family level. Violin plots show the distribution of relative abundances (log10-transformed) for the genera *Clostridium_Clostridiaceae* and *Haemophilus* and the families *Actinomycetaceae* and *Micrococcaceae*, stratified by DM status (noDM vs. DM). Boxplots within violins indicate median and interquartile range, with individual samples overlaid as points. Analyses were adjusted for age, sex, BMI, and GNRI. Only taxa passing the significance threshold (FDR < 0.05) and sensitivity checks are shown.

Differential abundance analysis at the genus level using ANCOM-BC2 identified two genera that were significantly associated with DM status after FDR correction (q < 0.05; Figure 5). Specifically, an unclassified Clostridiaceae taxon, *Clostridium_Clostridiaceae* (logFC = −0.123), and *Haemophilus* (logFC = −2.133) showed significant differences in relative abundance between individuals with and without DM, independent of age, sex, and BMI. No additional genera were significantly different after multiple-testing correction or when testing the interaction between DM status and the GNRI. Relative abundances of the significant genera are shown in Figure 5 on a log10 scale. The analyses were repeated at the family level, identifying two families that were significantly associated with DM status after FDR correction (q < 0.05; Figure 5). Specifically, *Actinomycetaceae* (logFC = 0.547) and *Micrococcaceae* (logFC = 2.433) were significantly different in relative abundance between individuals with and without DM, independent of age, sex, and BMI. No additional families were significantly differently distributed after multiple-testing correction or testing the interaction of DM status and the GNRI. Relative abundances of the significant families are shown in Figure 5 on a log10 scale.

## 4. Discussion

In summary, in this study, alpha diversity did not differ significantly between nursing-home residents with and without DM, and no interaction with the GNRI was observed. However, there were significant differences in beta diversity indices according to the GNRI, and differential abundance analysis revealed two genera and two families that were significantly associated with DM after adjustment for relevant covariates. These findings suggest that DM may be linked to specific compositional changes in the gut microbiota rather than broad shifts in overall microbial diversity. Nevertheless, concerning microbial diversity, malnutritional risk may represent a more relevant determinant than DM per se.

Our finding of no significant difference in alpha diversity in individuals with DM contrasts with several previous studies reporting differences between DM and individuals without DM, which may in part be explained by the observational and cross-sectional design of our study [27,28,29,30]. Previous investigations have described reduced richness or diversity in type 2 DM; others have failed to detect significant global diversity shifts, which might suggest that DM-related microbial alterations may be only small and rather cohort-specific, particularly after adjustment for relevant confounders such as BMI, diet, and medication use [6,27,28,29,30,31]. An additional explanation for the absence of significant alpha diversity differences in our cohort may be the widespread use of antidiabetic medication among residents with DM. Antidiabetic drugs, particularly metformin, are known to influence gut microbial composition and may partially attenuate or modify diabetes-associated microbiome signatures [32,33]. Consequently, medication effects could have reduced detectable differences between residents with and without DM. Although previous studies have not consistently reported changes in alpha diversity following metformin treatment, medication-related shifts in specific microbial taxa have been described, including changes in *Anaerostipes*, *Proteobacteria*, and *Verrucomicrobia* [32,33]. These taxa were not identified in our analysis. Nevertheless, it is known that type 2 DM is associated with profound gut microbiota dysbiosis characterized by decreased microbial diversity, reduced abundance of beneficial butyrate-producing bacteria (such as *Faecalibacterium prausnitzii* and *Roseburia*), and increased proinflammatory bacteria like *Escherichia-Shigella* [27,34,35]. Furthermore, the microbiota influences DM risk through multiple mechanisms, including production of short-chain fatty acids that regulate glucose homeostasis and insulin sensitivity, generation of bacterial metabolites that act as endocrine-like signals affecting host metabolism, and modulation of systemic inflammation [36,37]. In our study, participants with DM exhibited generally good glycemic control, as reflected by HbA1c levels, which may also have contributed to the absence of significant differences in alpha diversity. Bearing in mind the mean age of 86 years in our study cohort, a mean HbA1c level of 6.0% could also be caused by overtreatment. Previous literature already claimed that older individuals with DM might be overtreated [38]. Furthermore, the current literature claims that richness and the Shannon index might not be sensitive enough to measure subtle differences in alpha diversity in specific cohorts [27,30]. Therefore, analyses focusing on specific taxa or beta diversity are required to identify meaningful differences in microbial community composition. Furthermore, our study population had a mean age of over 80 years, raising the question of how alpha diversity still changes at an advanced age. Previous research suggests that gut microbial alpha diversity tends to decline with advancing age [9,39]. This age-related reduction may partly explain why no significant differences in alpha diversity were observed between individuals with and without DM in this older cohort. Nutrition, particularly dietary fiber intake, plays a critical modulatory role in the aforementioned bidirectional relationship by directly shaping gut microbiota composition and function, with dietary changes inducing shifts in gut microbiota within 24 h [36]. It is known that the GNRI reflects protein-energy status and reduced dietary intake, both of which directly alter the gut microbiota [40,41]. Studies have shown that older persons with malnutrition, e.g., secondary to reduced appetite, have significant reductions in alpha diversity and show depletions in beneficial taxa [12,13]. Recent evidence discovered links between elevated uric acid levels and higher IL-6 and IL-1β concentrations in older adults, highlighting the role of metabolic dysregulation in chronic low-grade inflammation [42]. Consequently, albumin and the GNRI may reflect inflammatory status in addition to nutritional status and should be interpreted accordingly [42]. In our study, PERMANOVA analyses based on Bray–Curtis distances showed that malnutritional risk was significantly associated with microbial community composition, whereas DM status was not associated with beta diversity after adjustment for age, BMI, sex, and nursing home. These findings suggest that, in this nursing-home cohort, malnutritional risk may play a more important role in shaping gut microbial community structure than DM status itself. Moreover, our cohort consisted of older adults living in nursing homes, in whom glycemic control was generally well managed or the individuals were even overtreated, as reflected by HbA1c levels that were largely comparable between individuals with and without DM. In this context, the absence of DM-associated differences in microbial community structure may indicate that well-controlled DM does not substantially alter the gut microbiota compared with individuals without DM. Instead, our results suggest that malnutritional risk may represent a more relevant factor in shaping microbial community composition in this population.

Regarding beta diversity, differences between individuals with and without DM in older populations were found. A previous Nigerian study found significant differences in gut microbiota of individuals with versus without DM aged 60 years or older [9]. *Clostridiaceae* and *Peptostreptococcaceae* were enriched in healthy individuals but depleted in those with DM [9]. Previous studies also link higher *Clostridiaceae* levels to better metabolic markers and report these families as dominant in healthy populations [43]. Similar findings were also reported in our study, with lower levels of *Clostridiaceae* in the DM versus the noDM cohort in individuals living in nursing homes. Still, no significant differences in *Peptostreptococcaceae* in our study were found. Furthermore, our study revealed DM status-related differences in *Actinomycetaceae* levels, which is a family within the Actinobacteria phylum. *Actinomycetaceae* levels vary with aging [44]. In healthy individuals over 80 years, their abundance may remain stable or slightly increase, whereas a reduction has been observed in individuals with DM [44]. *Actinomycetaceae* are involved in the production of short-chain fatty acids and in immune system regulation [45,46]. A decline of this group in DM and advanced age may contribute to reduced gut barrier function and increased susceptibility to inflammation [45,46]. Our study further reported DM status-related differences in *Haemophilus* and *Micrococcaceae*; however, no previous literature reported their effects in older individuals or in subjects with DM. Previous literature claims fewer differences in beta diversity related to age as aging is associated with a decline in microbial diversity [39,47]. Nevertheless, the functional capacity of the microbiota remains relatively conserved across healthy older individuals [39,47]. Age-related changes tend to be less pronounced than disease-related shifts, and interindividual variability increases with age, making age-associated beta diversity differences less distinct compared to those driven by DM, which might explain the few differences found in our study [39,47]. However, this underscores the need for age-matched controls in microbiota studies to accurately discern disease-specific alterations. While in our study some differences in microbial composition were observed according to DM status alone, these differences did not persist when examining the interaction between DM and GNRI on microbiota diversity. In other words, although DM may be associated with certain compositional shifts, it did not modify the relationship between malnutritional risk and the microbiota. The effect of the GNRI on microbial composition appeared comparable in older individuals with and without DM. One possible reason for this lack of interaction might be that residents of nursing homes are medically well cared for, receiving regular medical check-ups every couple of weeks. Still, previous studies have discussed how aging, frailty, multiple chronic diseases, and institutional living affect the gut microbiome of nursing-home residents. Therefore, changes in the gut microbiome may contribute to poorer health outcomes and that malnutrition, physical activity, and targeted interventions could help improve healthy aging and quality of life in this population [48]. Furthermore, recent evidence indicates that malnutrition in older adults is associated with alterations in biochemical, hematological, immunological, and oxidative stress markers, highlighting the complex interplay between nutritional status, inflammation, and metabolic dysregulation [49]. In addition, microbiome-derived short-chain fatty acids, particularly butyrate, play a crucial role in maintaining intestinal barrier integrity and regulating inflammation, obesity, and insulin resistance [50]. Together, these findings provide important clinical and mechanistic context for understanding the links between malnutrition, gut microbiota, and healthy aging. Another reason might be that malnutrition itself has a more pronounced effect on the microbiota, thus overshadowing modest DM-specific effects [51].

Several limitations of this study should be acknowledged. First, the overall sample size was relatively small, and the DM and noDM groups were not equally balanced, which may have limited statistical power to detect subtle differences. Also, the type of DM was not defined. Nevertheless, globally, type 2 DM accounts for the vast majority of DM cases, representing approximately 90–95% of all diagnosed DM worldwide [52]. Second, more than half of the participants were female, potentially limiting the generalizability of the findings and introducing sex-related bias in microbial composition. Third, the cross-sectional design of the study precludes any conclusions regarding causality and limits the ability to infer temporal relationships between DM status, nutritional status, and microbiota composition. The specific stool collection method was not systematically documented for individual participants. Therefore, potential effects of collection from incontinence pads/diapers versus disposable collection aids on microbiota composition could not be assessed and may have contributed to unmeasured variability in the results. Finally, individuals with DM in our cohort had generally good glycemic control, which may have attenuated disease-associated microbial alterations and reduced the likelihood of detecting stronger effects.

## 5. Conclusions

Taken together, our results suggest that in older adults, malnutritional risk may represent a more relevant determinant of gut microbial variation than DM per se. Although DM has been associated with microbiota alterations in other populations, malnutritional risk in our cohort showed a clearer association with microbial community composition, independent of DM status. These findings highlight the importance of maintaining adequate and balanced nutritional intake in older individuals, including those with DM, when considering factors that may influence gut microbial homeostasis.

## Figures and Tables

**Table 1:** Baseline characteristics.

Variable	NoDM (n = 122)	DM (n = 51)	*p*-Value
Age	85 (8.21)	83 (9.38)	0.200
Sex			0.900
female	95 (78%)	39 (76%)	
male	27 (22%)	12 (24%)	
BMI			0.064
underweight	9 (7.4%)	1 (2.0%)	
normal	57 (47%)	21 (41%)	
overweight	25 (20%)	8 (16%)	
obese	31 (25%)	21 (41%)	
HbA1c	5.53 (0.93)	6.19 (1.04)	0.002
GNRI			0.400
no risk	42 (40%)	23 (49%)	
risk	63 (60%)	24 (51%)	
Cardiovascular disease	59 (48%)	21 (53%)	0.600
High blood pressure	80 (66%)	35 (69%)	0.500
Cancer	24 (20%)	11 (22%)	0.700
Stroke	28 (23%)	10 (20%)	0.900
Neuropsychiatric disease	61 (50%)	16 (31%)	0.010
Gastrointestinal disease	39 (32%)	11 (22%)	0.400

Baseline characteristics of the study population stratified by diabetes mellitus status (DM versus NoDM). Continuous variables are presented as mean and standard deviation and categorical variables as n/N (%). *p*-values were calculated using Student’s *t*-test for continuous variables and the chi-square test for categorical variables. BMI = body mass index; HbA1c = glycated hemoglobin; GNRI = Geriatric Nutritional Risk Index.

## Data Availability

All sequence data from this project have been deposited in the Sequence Read Archive of the National Center for Biotechnology Information under BioProject accession number (PRJNA1473107).
